# Employee–Spouse Perceptual Congruence in Employee Work-to-Family Enrichment Affects Family and Work Outcomes: The Mediating Role of Relationship Conflict

**DOI:** 10.3389/fpsyg.2021.660987

**Published:** 2021-07-19

**Authors:** Jianlan Chen, Yu Tian

**Affiliations:** School of Business, Sun Yat-sen University, Guangzhou, China

**Keywords:** work-to-family enrichment, congruence, relationship conflict, family cohesion, emotional exhaustion

## Abstract

Various studies have demonstrated that work-to-family enrichment (WFE) benefits employees in both the work and home domains. However, these findings may overstate the benefits of WFE and ignore its potential dark side. We advance the research on WFE by integrating conflict theory into the concept of WFE to investigate whether and how employee–spouse perceptual congruence in employee WFE influences employee family cohesion and emotional exhaustion. The results of polynomial regressions on 225 employee and spouse dyads revealed that the perceptual congruence in employee WFE between employees and spouses was negatively associated with relationship conflict. Additionally, asymmetrical incongruence effects were found, wherein spouses perceived a higher relationship conflict with employees when their perceptions of employee WFE were lower than those of the employees. Furthermore, spouses' perceived relationship conflict with employees mediated the influences of employee–spouse perceptual congruence in employee WFE on employee family cohesion and emotional exhaustion.

## Introduction

With the development of both the positive psychology movement (Seligman, 2002) and the positive organizational behavior movement (Luthans, 2002), positive relationships between work and family roles have attracted the interest of researchers. Work-to-family enrichment (WFE) describes the positive relationships between work and family roles and is defined as the extent to which experiences in work roles improve the quality of life in family roles (Greenhaus and Powell, 2006). Under this definition, a wide body of evidence from numerous studies indicates that high WFE levels have positive family and work effects, including higher family and job satisfaction, higher family functioning, and higher job performance (Mcnall et al., 2010; Carlson et al., 2011, 2019; Oren and Levin, 2017; Wayne et al., 2017, 2020; Zhang et al., 2018; Kalliath et al., 2020; Wu et al., 2020).

Although many studies have found that WFE benefits the work and family life of employees, to the best of our knowledge, no research has been published on its potential drawbacks. In this study, we showed that scholars may have overstated the benefits of WFE and overlooked its costs. For example, it is possible that employees who perceive high levels of WFE may not always have positive experiences with their family members. Most prior studies about WFE of employees used only self-reported measures to assess the WFE of the employees (Mcnall et al., 2010) and contained a prevailing and unproven assumption that employees perceived WFE to be accurate and objective, which means that if the employees perceive high WFE, then their family members also experience it and benefit greatly from their work (e.g., Carlson et al., 2009; Liao et al., 2016). However, “self-reports at best capture individuals' perceptions of enrichment rather than enrichment *per se*” (Greenhaus and Powell, 2006, p. 87). Indeed, correlations between self-rated and spouse-rated work-to-family spillover were relatively low (e.g., Grandey et al., 2005; Ilies et al., 2007), signifying that they measure similar but unique experiences. Thus, it is possible that when employees perceive high WFE, their family members may not also experience it. Moreover, this kind of perceptual difference will result in relationship conflicts between employees and their family members and lead to negative family and work effects according to conflict theory (De Dreu and Weingart, 2003; De Dreu, 2008).

**Figure 1:**
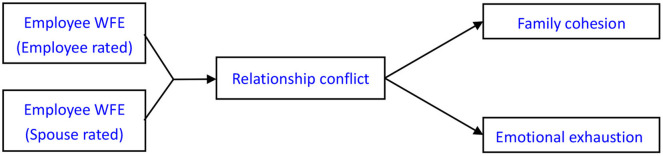
Theoretical model for the current research.

To address these questions, we draw on conflict theory and the WFE literature to study whether and how employee–spouse (The present research chose and investigated spouses of the employees as representatives of their family members.) perceptual (in)congruence in WFE of the employees predicts family and work consequences. The conflict theory notes that real or perceived differences between individuals will result in relationship conflict (i.e., tension and friction), which in turn may lead to negative outcomes. We discussed that dyadic incongruence in perceived employees' WFE represents a certain existing difference between employees and their spouses, thus increasing the perceptions of the spouses on relationship conflict with employees. We further propose that a higher spousal perception of relationship conflict with employees induced by dyadic perceptual incongruence in WFE, in turn, influences two main family and work consequences—family cohesion and emotional exhaustion (see Figure 1).

The present study makes several theoretical contributions. First, we contributed to the WFE literature by challenging the prevailing consensus that high WFE is universally positive, which reveals that scholars should simultaneously consider how the WFE is perceived by both the employees and the spouses. The extant research has largely shown that WFE of the employees predicts positive family and work effects (Mcnall et al., 2010) but ignores the potential dark side of their WFE. This study thus provides a more holistic theoretical understanding of the consequences of WFE of the employees. Second, we contribute to the WFE literature by examining a relationship-based mechanism between WFE and family/work consequences. Most research examining the relationships between WFE and outcomes did not include any mediator (Greenhaus and Powell, 2006). In the present study, we identify and examine relationship conflict as the mediator between WFE and family/work consequences and thus offer a new relational mechanism that links WFE and outcomes. Finally, this study extends the family cohesion and emotional exhaustion literature by examining employee–spouse perceptual (in)congruence in WFE of the employees as antecedents of both family cohesion and emotional exhaustion. Prior studies have examined the impact of the perceptions of both employees and spouses on the WFE of the employees on family and work variables separately (e.g., Oren and Levin, 2017; Zhou et al., 2018; Tan et al., 2021) while overlooking their joint impact.

### Theory and Hypothesis Development

#### Employee–Spouse Perceptual Congruence in the WFE of Employees and Relationship Conflict

Perceptual congruence refers to the degree of overlap or agreement in perceptions of the same stimulus (Benlian, 2013, 2014). Scholars believe that the perceptual process in humans is very complex, and each person's complex perceptual process is determined by his or her own characteristics, including personality, values, and experience (Allport, 1955; Benlian, 2013, 2014). Therefore, perceptual incongruence between people is very common, even in intimate relationships (e.g., couple relationships).

Relationship conflict has been defined as “an awareness of interpersonal incompatibilities” (Jehn and Mannix, 2001, p. 238). People who perceive a high level of relationship conflict with someone will dislike him or her and feel tension, friction, annoyance, frustration, and irritation toward that person (Jehn and Mannix, 2001; Graham et al., 2018). In the current study, the perceptions of spouses on the relationship conflict with employees reflect the spouses' perceived relationship quality with employees.

Using the conflict theory tenet that real or perceived differences between individuals will result in relationship conflict (De Dreu and Weingart, 2003; De Dreu, 2008), we propose that dyadic perceptual incongruence in the WFE of employees will result in a worse relationship between spouses. In other words, we propose that as perceptual incongruence in WFE of employees increases between spouses—that is, moves from dyadic perceptual congruence conditions (e.g., high–high vs. low–low) toward dyadic perceptual incongruence conditions (e.g., high–low vs. low–high)—spouses will perceive a higher level of relationship conflict with employees.

According to the conflict theory, the negative consequences of perceptual incongruence can be explained in two ways (Mohammed and Angell, 2004). One way is “self-categorization,” which suggests that to maintain their social identities, individuals embrace people who appear to have characteristics similar to those of their “in-group” members and to treat them positively (Tajfel and Turner, 1986). Conversely, individuals exclude people who have dissimilar characteristics as “out-group” members and treat them negatively (Turner et al., 1979; Ashforth and Mael, 1989). Another theoretical approach is “similarity–attraction,” which shows that individuals dislike anyone who happens to be unlike them. Individuals are more likely to be drawn to others who are more similar to them to reinforce their own values and beliefs (Byrne, 1971). Therefore, employee–spouse relationship quality depends on employee similarity. Take, for instance, a condition where an employee perceives a high WFE level and believes that he/she has made great contributions to his or her family. If his or her spouse perceives a low level of employee WFE, the employee–spouse dyad encounters perceptual incongruence in their interactions, potentially triggering tension and friction in the dyad, which results in relationship conflict. Conversely, when an employee perceives a high WFE level and his or her spouse also perceives a high employee WFE level, spouses feel more comfortable in dyadic interactions with employees and perceive a low level of relationship conflict with them.

A review of the literature reveals that perceptual incongruence between spouses is an important antecedent of marital relationship quality in couple relationship research (Iafrate et al., 2012; Young et al., 2014). In addition, previous research has supported this idea, showing that the dissimilarity between how people perceive themselves (in terms of their contributions, strengths, and so on) and how others perceive them may decrease interpersonal attraction and liking and increase interpersonal conflict (Swann et al., 1992; Polzer et al., 2002; Qin et al., 2019). We thus predict that the perceptions of spouses on relationship conflict with employees will increase when perceptions of WFE of employees are misaligned and will decrease when they become aligned.

*H1: More incongruent employee and spouse levels of perceived employee WFE are associated with higher spouse perceptions of relationship conflict with employees*.

#### Differentiating the Two Types of Employee–Spouse Perceptual Congruence

Employees and spouses can be congruent at either high or low levels of WFE ratings. We posit that the perceptions of the spouse on the relationship conflict between them will be lower in the condition of high–high WFE ratings compared with that of the low–low WFE ratings because employees with high levels of WFE ratings are more likely to invest energy and effort into improving the quality of life in the family domain, such as by offering care, warmth, and safety to their family members (i.e., spouse) (Bakker and Geurts, 2004; Greenhaus and Powell, 2006). Additionally, spouses with high levels of WFE ratings are more likely to acknowledge and appreciate the focus and effort that employees contribute to the family (Ten Brummelhuis and Bakker, 2012). Therefore, in the condition of high–high WFE ratings, the energy and efforts being made by the employees in their family role and the same being acknowledged and appreciated with regard to their focus and effort, lead to supportive mutual understanding and help to enhance mutual relationship maintenance between employees and their spouses (Bakker and Geurts, 2004; Greenhaus and Powell, 2006), which in turn can reduce relationship conflict.

In comparison, when employees and spouses are congruent at low levels of WFE ratings, although they reach an agreement on employee WFE and experience fewer relationship conflicts, employees are less likely to put forth effort and energy in improving their family quality of life, and their spouses are less likely to acknowledge and appreciate their focus and effort (Bakker and Geurts, 2004; Greenhaus and Powell, 2006). Therefore, in the condition of low–low WFE ratings, employees and spouses are less likely to have a supportive mutual understanding and help each other enhance mutual relationship maintenance compared with those in the condition of high–high WFE ratings. Thus, we propose the following:

*H2: Spouses' perceptions of relationship conflict with employees are lower when employees are congruent with spouses at high levels of perceived employee WFE than when employees are congruent with spouses at low levels of perceived employee WFE*.

#### Differentiating Between the Two Types of Employee–Spouse Perceptual Incongruence

Employees and spouses can be incongruent at all levels of WFE ratings, and we posit that perceptions of spouses regarding relationship conflict will be weaker in the condition of low–high WFE ratings than in that of high–low WFE ratings. Employees with high levels of WFE ratings are more likely to invest energy and effort into improving the quality of life at home by offering care, warmth, and safety to their spouses (Bakker and Geurts, 2004; Greenhaus and Powell, 2006; Ten Brummelhuis and Bakker, 2012). However, spouses with low levels of WFE ratings are less likely to acknowledge and appreciate the focus and effort of the employees (Ten Brummelhuis and Bakker, 2012). More importantly, previous studies on self-perception highlighted that own perception of an individual is more important than that of others (Kahn, 1990), as the experience of the individual is filtered through his or her own perception and cognition (Matta et al., 2015). Thus, the benefits resulting from high levels of WFE of employees are less likely to mitigate relationship conflict if spouses believe that the WFE of employees is low.

Alternatively, in the condition of low–high WFE ratings, the lack of benefits related to a low level of employee WFE is likely to be less detrimental to relationship conflict if the spouse thinks that the WFE of the employee is high. In this case, although the spouse may perceive relationship conflict resulting from WFE incongruence, the spouse may also appreciate the efforts of the employee toward improving the quality of life of the family. That is, the acknowledgment and appreciation by the spouse driven by his/her perception of high levels of WFE can alleviate relationship conflict resulting from WFE incongruence. As a result, the perception of the spouse on relationship conflict will be reduced. Accordingly, we propose that the perception of the spouse on relationship conflict will be lower when his/her rating of WFE is higher, rather than when it is lower, than that of the employee, yielding the following hypothesis:

*H3: A spouse's perceptions of relationship conflict with an employee are lower when his/her perception of employee WFE is higher than the employee's own perception compared to when the spouse's perception of employee WFE is lower than the employee's own perception*.

#### Relationship Conflict as a Mediator of the (in)congruence Effect on Family and Work Outcomes

According to the literature, family cohesion has been defined as the emotional bonding that exists among family members (Olson et al., 1983). When family cohesion is high, family members have a great amount of emotional closeness and dependence, whereas when family cohesion is low, a great deal of personal separateness and independence is found among family members (Olson et al., 1983). A high level of relationship conflict often induces tension, friction, annoyance, frustration, and irritation between the two sides (Jehn and Mannix, 2001; Graham et al., 2018). Therefore, we propose that the tension and negative emotions elicited by relationship conflict are negatively associated with family cohesion, as they will lead to personal separateness and little involvement among both parties in the dyad. The results of this empirical study also support our hypothesis. For example, a prior study found that low levels of family cohesion are correlated with high levels of psychological distress (Fujiwara et al., 2003).

In addition, negative interpersonal experiences, such as interpersonal conflict, are characterized by tension and stress. Additionally, high levels of relationship conflict engender an unpleasant and stressful family environment, which produces a great deal of psychological distress (Leiter and Maslach, 1988; Fujiwara et al., 2003). Employees must exert greater effort to cope with these stresses (Oren and Levin, 2017), which in turn leads to emotional exhaustion (Alarcon, 2011). Moreover, interpersonal conflict limits the information-processing abilities of the employees, and the existing literature has demonstrated that emotional exhaustion results from using inadequate strategies to cope with problematic interpersonal events (Alarcon, 2011). Empirically, researchers have found that negative interpersonal interactions are potentially stressful and are directly associated with higher levels of emotional exhaustion (Leiter and Maslach, 1988).

Given that we have hypothesized the effects of employee–spouse perceptual (in)congruence in employee WFE on the perceptions of spouses on the relationship conflict with employees (i.e., Hypothesis 1) and the established relationship between relationship conflict and employee family and work outcomes, we expected that relationship conflict carries congruence effects on employee family cohesion and emotional exhaustion. Thus, we formulated the following two hypotheses about the mediating role for relationship conflict:

*H4: A spouse's perceptions of relationship conflict with an employee mediate the relationship between employee–spouse (in)congruence in perceived employee WFE and family cohesion*.
*H5: A spouse's perceptions of relationship conflict with an employee mediate the relationship between employee–spouse (in)congruence in perceived employee WFE and emotional exhaustion*.

## Methods

### Participants and Procedure

We contacted our participants through the alumni networks of several large universities in southern China. This method allowed us to invite the participation of employees working in various industries and positions, which enhanced the external validity of the findings (Qin et al., 2018). We initially invited 258 pairs of employees (who were married and worked full-time) and their spouses to participate in our survey. We sent links to the surveys to all the employees and their spouses *via* WeChat or email. Employees were asked to complete a questionnaire that included measures of their demographic information, WFE, and emotional exhaustion. Spouses were asked to complete questionnaires that included measures of their demographic information, relationship conflict with employees, family cohesion, employee work–family conflict (WFC) and employee WFE. Ultimately, we collected 225 dyadic data points matching employee and spouse responses (with a response rate of 87.2%).

Among the 225 employees, 50.7% were female, and their average age was 34.52 years (*SD* = 7.89). Of the employees, 40.4% received college degrees or lower, 52.9% received bachelor's degrees, 5.8% received master's degrees, and 0.9% received doctoral degrees. The mean age of the spouses was 34.54 years (*SD* = 7.75); of them, 42.2% received college degrees or lower, 47.6% received bachelor's degrees, 7.1% received master's degrees, and 3.1% received doctoral degrees.

### Measures

#### WFE

WFE was assessed using a four-item scale developed by Grzywacz and Marks (2000). Employees assessed their own WFE using self-reported items. A sample item was as follows: “The things you do at work help you deal with personal and practical issues at home.” Spouses were provided with four WFE items similar to the self-reported items and were asked to describe their spouses. A sample item was as follows: “The things he/she does at work help him/her deal with personal and practical issues at home.” Both employees and their spouses indicated their responses on a five-point Likert scale (1 = strongly disagree; 5 = strongly agree). The coefficient α was 0.706 for employees and 0.804 for spouses.

#### Relationship Conflict

To measure relationship conflict, we selected three items related to spousal relationships from the intragroup conflict scale (Jehn, 1995) and adapted them to better suit the subjects of the current study. Spouses rated their level of relationship conflict with their spouses on a four-point scale ranging from 1 (almost never) to 4 (a lot). An example item was as follows: “How much emotional conflict exists between you and your spouse?” The coefficient α for this scale was 0.611.

#### Family Cohesion

Family cohesion was measured using four items derived from the Family Adaptability and Cohesion Evaluation Scale (FACES III, Olson, 1986). The original scale for family cohesion had 10 items from which we took the four highest loading items. An example item was as follows: “Our family does things together.” Spouses were asked to evaluate their family cohesion on a five-point scale ranging from 1 (never) to 5 (always). The coefficient α for this scale was 0.841.

#### Emotional Exhaustion

Employees assessed their own emotional exhaustion using Schaerer et al. (2018) three-item emotional exhaustion scale. The scale was measured using a five-point Likert format (1 = strongly disagree; 5 = strongly agree). An example item was as follows: “I feel emotionally drained from my work.” The coefficient α for this scale was 0.745.

#### Control Variables

Prior research has suggested that demographic (dis)similarities may relate to interpersonal relationships (Tsui et al., 2002; Levin et al., 2006). Aligning with prior interpersonal relationship research (e.g., Zhang et al., 2012; Graham et al., 2018), we controlled for age dissimilarity and education dissimilarity when testing our predictions. Specifically, with regard to age dissimilarity, the absolute value of their difference was calculated. Additionally, education (1 = college degree or lower, 2 = bachelor's degree, 3 = master's degree, and 4 = doctoral degree) dissimilarity was operationalized as the absolute value of their difference. We also controlled for the gender of the employees instead of gender dissimilarity because all of the participants were heterosexual couples. Furthermore, WFC was included as a control variable because it has been meta-analytically associated with marital satisfaction (Amstad et al., 2011). We controlled for employees' WFC using four items developed by Grzywacz and Marks (2000; α = 0.864).

### Analysis

We employed polynomial regression and response surface methodology to test Hypotheses 1–3 (Edwards and Parry, 1993). Polynomial regression has been well-developed and advocated for generating three-dimensional response surfaces and examining congruence effects on outcome variables (Edwards and Parry, 1993; Edwards and Cable, 2009). The polynomial regression equation is as follows (all control variables are omitted for simplicity):

(1) $$\begin{array}{l} R=b_{0}+b_{1}E+b_{2}S+b_{3}E^{2}+b_{4}E\times S+b_{5}S^{2}+e \end{array}$$

where *R* represents relationship conflict, *E* represents employee-rated WFE, and *S* represents spouse-rated WFE. To eliminate multicollinearity and facilitate interpretation of the results, we mean-centered employee and spouse WFE before calculating the second-order terms (Wilson et al., 2018). After the polynomial regressions were conducted, we plotted the three-dimensional response surfaces with the regression coefficients.

To test Hypothesis 1 (i.e., congruence effect between an employee and his/her spouse), we first tested the joint significance of the coefficients for three second-order terms (*E*^2^, *E* × *S*, and *S*^2^). Furthermore, we tested two features of the plotted three-dimensional response surface, as suggested by Edwards and Cable (2009). The first feature is the curvature along the incongruence line (*E* = -*S*). To support Hypothesis 1, the curvature along the incongruence line (calculated as *b*_3_ - *b*_4_ + *b*_5_) must be significant and positive. The second feature is the bottom, where values for the dependent variable are minimized. To provide additional support for Hypothesis 1, the bottom of the response surface should run along the congruence line, which would show that the values for relationship conflict are minimized when the values for employee and spouse WFE are congruent. To achieve this condition, the second principal axis of the response surface should have the slope *p*_21_ = 1 and intercept *p*_20_ = 0 (Edwards and Parry, 1993). Consistent with prior studies using polynomial regression and response surface methodology (e.g., Edwards and Cable, 2009; Wilson et al., 2018), 10,000 bootstrapped samples were conducted to estimate 95% confidence intervals (CIs) for *p*_21_ and *p*_20_ (Edwards and Parry, 1993).

For Hypothesis 2, we examined the line slope (calculated as *b*_1_ + *b*_2_) of the response surface of relationship conflict along the congruence line (*E* = *S*). To support Hypothesis 2, the congruence line slope must be significant and negative. To test Hypothesis 3 (the asymmetrical incongruence effect), we generated 10,000 bootstrapped samples to estimate 95% CIs for the lateral shift quantity (calculated as (*b*_2_ – *b*_1_)/[2 (*b*_3_ – *b*_4_ + *b*_5_)]). The value for the lateral shift quantity indicates the magnitude and direction of the lateral shift in the response surface along the incongruence line, so a negative value for the lateral shift quantity would support Hypothesis 3.

To test the indirect effect of employee–spouse congruence on family cohesion and emotional exhaustion *via* relationship conflict (Hypotheses 4 and 5), we utilized the block variable approach (Edwards and Cable, 2009). First, we combined the five polynomial regression terms (*E, S, E*^2^, *E* × *S*, and *S*^2^) into a block variable based on their respective weights in the polynomial regression model. Second, we regressed the mediator (i.e., relationship conflict) on the block variable to obtain a single coefficient (γ_1_), representing the joint effect of the five terms on relationship conflict. We then regressed family cohesion and emotional exhaustion on both relationship conflict and the block variable to obtain the coefficients representing the effects of relationship conflict on family cohesion (γ_2_) and emotional exhaustion (γ_3_). Finally, we examined the significance of the indirect effects (γ_1_ × γ_2_ and γ_1_ × γ_3_) using bootstrapping.

## Results

Table 1 presents the mean, standard deviations, and correlations of the variables. Employee-rated WFE is correlated with emotional exhaustion (*r* = −0.201, *p* < 0.01). Spouse-rated employee WFE is correlated with relationship conflict (*r* = −0.200, *p* < 0.01) and family cohesion (*r* = 0.313, *p* < 0.01). Relationship conflict was correlated with family cohesion (*r* = −0.278, *p* < 0.01) and emotional exhaustion (*r* = 0.143, *p* < 0.05).

**Table 1:** Means, standard deviations, and correlations among variables.

**Variable**	***M***	***SD***	**1**	**2**	**3**	**4**	**5**	**6**	**7**	**8**	**9**
1. Gender	0.510	0.501									
2. Age dissimilarity	2.244	3.401	−0.005								
3. Education dissimilarity	0.333	0.526	0.017	0.328^**^							
4. Employee WFC	2.459	0.921	−0.183^**^	−0.055	−0.076						
5. Employee WFE (employee–rated)	3.387	0.828	0.085	−0.051	−0.131^*^	−0.025					
6. Employee WFE (spouse-rated)	3.900	0.821	0.126^†^	0.094	0.121^†^	−0.073	−0.053				
7. Relationship conflict	1.113	0.321	0.005	−0.017	0.111^†^	0.136^*^	0.105	−0.200^**^			
8. Family cohesion	3.914	0.762	0.038	0.053	0.035	−0.207^**^	−0.015	0.313^**^	−0.278^**^		
9. Emotional exhaustion	3.714	0.627	−0.067	0.077	−0.026	0.062	−0.201^**^	−0.085	0.143^*^	−0.002	

*N = 225.*

† *p < 0.10.*

* *p < 0.05.*

** *p < 0.01*.

Prior to testing our hypotheses, we conducted a series of confirmatory factor analyses to confirm that the constructs assessed in this study could be distinguished from one another. As Table 2 shows, our proposed four-factor model was a better fit for the data (χ^2^ = 75.980, *df* = 71, comparative fit index = 0.994, Tucker–Lewis index = 0.992, root mean square error of approximation = 0.018, and standardized root mean square residual = 0.044) than the two three-factor models, the two-factor model and the single-factor model. Thus, the results indicated that the four constructs could be distinguished from one another.

**Table 2:** Model fit results for confirmatory factor analyses.

**Models**	**χ^**2**^**	***df***	**Δχ^**2**^(**Δ***df*)**	***CFI***	***TLI***	***RMSEA***	***SRMR***
Four-factor: WFE; RC; FC; EE	75.980	71		0.994	0.992	0.018	0.044
Three-factor: WFE; RC + FC; EE	163.159	74	87.179^**^ (3)	0.892	0.867	0.073	0.071
Three-factor: WFE + EE; RC; FC	224.496	74	148.516^**^ (3)	0.818	0.776	0.095	0.087
Two-factor: WFE + EE; RC + FC	302.536	76	226.556^**^ (5)	0.726	0.672	0.115	0.099
Single-factor: WFE + EE + RC + FC	690.926	77	614.946^**^ (6)	0.257	0.121	0.188	0.174

*N = 225.*

** *p < 0.01. All alternative models were compared with the hypothesized four-factor model. CFI, comparative fit index; TLI, Tucker–Lewis index; RMSEA, root mean square error of approximation; SRMR, standardized root mean square residual; WFE, work-to-family enrichment; RC, relationship conflict; FC, family cohesion; EE, emotional exhaustion*.

**Figure 2:**
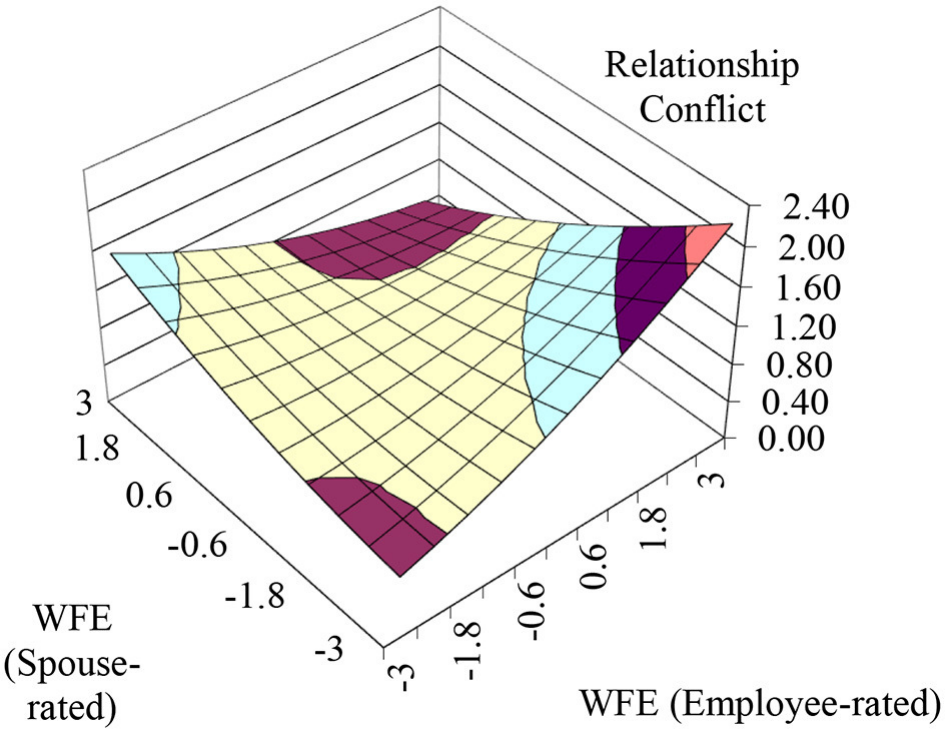
The congruence effects of employee-rated and spouse-rated employee WFE on relationship conflict.

Hypothesis 1 predicted that the more aligned the employee- and spouse-rated WFE levels are, the lower the perception of the spouse on the relationship conflict with the employee will be. To test Hypothesis 1, we conducted polynomial regressions, and the results are shown in Table 3. The results in Model 2 showed that the three second-order polynomial terms were jointly significant (*F* = 2.663, *p* < 0.05), and the curvature along the incongruence line was significant and positive (curvature = 0.109, *p* < 0.01). Furthermore, the results of the bootstrapping analyses showed that the second principal axis had a slope (*p*_21_) that did not significantly differ from 1.0 [95% CI (0.515, 8.694), containing 1] and an intercept (*p*_20_) that did not significantly differ from zero [95% CI (−0.036, 6.809), containing zero]. To visually present these results, we plotted the overall response surface in Figure 2 based on the coefficients in Model 2. The convex surface implies that the relationship conflict levels between employees and their spouses decrease as the employee- and spouse-rated employee WFE levels become more aligned, and the relationship conflict levels between employees and their spouses increase as the employee- and spouse-rated employee WFE levels become more discrepant. As a result, Hypothesis 1 is supported.

**Table 3:** Polynomial regressions of relationship conflict, family cohesion, and emotional exhaustion on WFE congruence.

**Variables**	**Relationship conflict**		**Family cohesion**		**Emotional exhaustion**	
	**M1**	**M2**	**M3**	**M4**	**M5**	**M6**
Constant	0.949^**^ (0.070)	0.918^**^ (0.077)	4.027^**^ (0.174)	4.479^**^ (0.219)	3.676^**^ (0.154)	3.414^**^ (0.197)
**Controls**						
Gender	0.028 (0.043)	0.039 (0.043)	−0.016 (0.096)	0.003 (0.094)	−0.036 (0.085)	−0.047 (0.085)
Age dissimilarity	−0.004 (0.006)	−0.004 (0.006)	0.007 (0.014)	0.005 (0.014)	0.017 (0.013)	0.019 (0.013)
Education dissimilarity	0.107^*^ (0.043)	0.105^*^ (0.042)	−0.053 (0.094)	−0.001 (0.093)	−0.082 (0.083)	−0.112 (0.084)
WFC	0.050^*^ (0.023)	0.050 (0.024)	−0.084 (0.053)	−0.059 (0.053)	0.010 (0.048)	−0.004 (0.048)
**Polynomial terms**						
Employee WFE (*E*)	0.044 (0.025)	0.048 (0.026)	0.032 (0.059)	0.056 (0.058)	−0.155^**^ (0.052)	−0.169^**^ (0.052)
Spouse WFE (*S*)	−0.081^**^ (0.026)	−0.087^**^ (0.029)	0.427^**^ (0.066)	0.384^**^ (0.066)	−0.111 (0.058)	−0.086 (0.059)
*E*^2^		0.019 (0.024)	−0.061 (0.055)	−0.051 (0.054)	0.058 (0.049)	0.053 (0.048)
*E* × *S*		−0.076 (0.029)^**^	0.072 (0.065)	0.034 (0.065)	−0.112 (0.058)	−0.090 (0.058)
*S*^2^		0.014 (0.022)	0.221^**^ (0.051)	0.228^**^ (0.049)	−0.038 (0.045)	−0.042 (0.044)
**Mediator**						
Relationship conflict				−0.493^**^ (0.150)		0.285^*^ (0.135)
**Variance explained**						
*R*^2^	0.093^**^	0.124^**^	0.179^**^	0.215^**^	0.088^*^	0.107^**^
Δ*R*^2^	0.093^**^	0.031^*^	0.179^**^	0.038^**^	0.088^*^	0.019^*^
**Congruence line (*****E*** **=** ***S*****)**						
Slope (*b*_1_ + *b*_2_)		−0.039	0.459^**^	0.440^**^	−0.266^**^	−0.255^**^
Curvature (*b*_3_ + *b*_4_ + *b*_5_)		−0.043	0.232^**^	0.211^*^	−0.092	−0.079
**Incongruence line (*****E*** **=** **–*****S***)						
Slope (*b*_1_ – *b*_2_)		0.135^**^	−0.395^**^	−0.329^**^	−0.044	−0.083
Curvature (*b*_3_ – *b*_4_ + *b*_5_)		0.109^**^	0.088	0.142	0.132	0.101
*F* for the 3 quadratic terms		2.663^*^	7.214^**^		1.967	

*N = 225. Unstandardized regression coefficients and standard errors are reported.*

† *p < 0.10.*

* *p < 0.05.*

** *p < 0.01*.

Hypothesis 2 suggested that relationship conflict is lower when perceptual congruence between an employee and a spouse of employee WFE occurs at higher rather than lower employee WFE levels. Model 2 of Table 3 shows that the slope and curvature of the congruence line are both not significant (slope = −0.039, *ns*, curvature = −0.043, *ns*). These results indicate that the surface is flat along the congruence line. Thus, Hypothesis 2 is not supported.

Hypothesis 3 predicted an asymmetrical incongruence effect such that the perceptions of the spouses on the relationship conflict with employees are lower when their perceptions of employee WFE are higher than own perceptions of the employees compared to when the perceptions of spouses on employee WFE are lower than own perceptions of the employees. The lateral shift quantity was significantly negative [−0.843, 95% CI (−188.497, −0.488)], indicating a shift toward the region where *S* is greater than *E*. Thus, Hypothesis 3 is supported.

Hypotheses 4 and 5 predicted the mediating effect of relationship conflict on the relationship between perceptual congruence and family and work outcomes. As displayed in Table 4, the block variable for perceptual congruence between an employee and a spouse regarding the WFE of an employee was associated with relationship conflict (γ_1_ = 1.010, *p* < 0.01). Relationship conflict was related to family cohesion (γ_2_ = −0.554, *p* < 0.05) and emotional exhaustion (γ_3 =_ 0.294, *p* < 0.05). The bootstrapping analysis results showed that the indirect effects of employee–spouse congruence on family cohesion [γ_1_ × γ_2_ = −0.560, 95% CI (−1.122, −0.126)] and emotional exhaustion [γ_1_ × γ_3_ = 0.297, 95% CI (0.070, 0.692)] *via* relationship conflict are both significant, supporting Hypotheses 4 and 5.

**Table 4:** Results from tests of direct and indirect effects of congruence/incongruence in WFE on relationship conflict, family cohesion, and emotional exhaustion.

**Variables**	**Relationship conflict**	**Family cohesion**	**Emotional exhaustion**
Block variable	1.010** (0.277)	−1.326* (0.661)	0.067 (0.499)
Relationship conflict		−0.554* (0.236)	0.294* (0.120)
Indirect effect		−0.560*	0.297*
95% Bootstrapped bias-corrected CIs for the indirect effect		[−1.122, −0.126]	[0.070, 0.692]

N = 225. Unstandardized regression coefficients and standard errors are reported. All control variables are omitted for simplicity. ^*^p < 0.05. ^**^p < 0.01.

## Discussion

In this study, grounded in conflict theory, we tested the impact of the congruence of perceived employee WFE between the employee and his/her spouse on work and family outcomes, including family cohesion and emotional exhaustion, through relationship conflict. Using a multisource study, we found that employee–spouse perceptual congruence between employees and their spouses of employee WFE was associated with lower levels of employee–spouse relationship conflict, a greater level of family cohesion and reduced emotional exhaustion, suggesting the benefits of employee–spouse perceptual congruence. More interestingly, the results revealed asymmetrical incongruence effects: perceptions of spouses on relationship conflict with employees are lower when their perceptions of employee WFE are higher than own perceptions of employees compared to when perceptions of spouses on employee WFE are lower than own perceptions of employees. Furthermore, we found that perceptions of spouses on relationship conflict mediated the impact of perceptual congruence between employees and spouses of employee WFE on employee family and work outcomes. These findings challenge the prevailing consensus that high WFE is universally positive and show the need for scholars to simultaneously consider perceived employees' WFE of both employees and spouses.

### Theoretical Implications

This study has important theoretical significance for related research on WFE, conflict theory, family cohesion, and emotional exhaustion. First, this study explored the potential negative effects of WFE from the perspective of employee–spouse congruence, challenging the prevailing assumption that WFE is always good for employees. The extant research shows that WFE positively influences employee work and family outcomes, including family and job satisfaction, family functioning, and job performance of the employees (Mcnall et al., 2010; Carlson et al., 2011, 2019; Wayne et al., 2017; Zhang et al., 2018). However, the current research on WFE influence is extremely unbalanced, and extensively exploring its positive effects may lead both researchers and practitioners to overpraise its positive impact and ignore its potential dark side. Grounded in conflict theory by introducing the employee–spouse congruence perspective, our study explored the influence of employee–spouse WFE (in)congruence on husband–wife relationship conflict, employee family cohesion, and emotional exhaustion. Our study reveals that when employees and spouses are congruent in their perceived WFE, the relationship conflict the spouse perceives is significantly reduced, family cohesion is strengthened, and emotional exhaustion is reduced; when employees and spouses are incongruent in their perceived WFE, differences in perceived WFE levels are found that lead to relationship conflict, reduce family cohesion, and enhance emotional exhaustion. Thus, in general, through the employee–spouse congruence perspective, this study provides a more comprehensive and dialectical perspective for the impact of WFE on employees, especially its negative impact.

Second, this study also explores the influence of the mechanism of WFE on the work and family outcomes of the employees. Although many studies have analyzed the outcome variables of WFE, providing substantial evidence for the positive effects of WFE on the work and family of the employees (Mcnall et al., 2010), most of these studies did not include any mediator (Greenhaus and Powell, 2006). In this study, the relationship between WFE and outcome variables is explained from the perspective of relationship conflict, which provides new theoretical insights into the WFE literature from the relationship conflict perspective.

Third, this study also expands the concept of conflict theory. Conflict theory compares only the differences in the relationship conflict level between individuals under the two situations of congruence and incongruence, holding that congruence between individuals will reduce relationship conflict (De Dreu and Weingart, 2003; Mohammed and Angell, 2004). However, in addition to testing the simple congruence effect (i.e., congruence *vs*. incongruence), this work further discusses the asymmetric congruence effect of high–high WFE and low–low WFE and examines the asymmetrical incongruence effect of high–low WFE and low–high WFE. We offer an elaborative theory explaining why, compared to those in low–low congruent dyads, employee relationship conflict is lower in high–high congruence dyads and why employees experienced the highest level of relationship conflict when their perceived WFE was lower than that of their spouses. These comparisons provide a more nuanced understanding of the congruence and incongruence effects of employee–spouse WFE in general, thus broadening conflict theory with a more detailed derivation and empirical analysis of different situations.

Fourth, this study contributes to the literature regarding the exploration of the predictive value of family cohesion and emotional exhaustion. The majority of the extant literature proposes that the positive work state of employees often leads to high family cohesion and low emotional exhaustion (Stevens et al., 2002, 2006; Bekker et al., 2005; Montgomery et al., 2006). This study suggests that even if the employee has a positive work state (e.g., employee WFE), if it fails to reach congruence with that of the spouse (e.g., employee WFE as perceived by the spouse), it can also lead to negative family outcomes (e.g., low family cohesion) and work outcomes (e.g., high emotional exhaustion). Thus, our work enriches the literature on the antecedents of employee family cohesion and emotional exhaustion and introduces the boundary conditions that induce family cohesion and reduce emotional exhaustion, providing an important theoretical and empirical reference for future research.

### Practical Implications

Our research highlights that the match between a spouse's perceived employee WFE and own perception of the employees on their WFE critically impacts both the family and work domains. The findings of our research have several practical implications for both supervisors and employees.

First, from the perspective of supervisors, our research showed that incongruence in perceived employees' WFE can lead to emotional exhaustion. Our research can inform supervisors about the importance of complete understanding by spouses regarding the employee WFE. This incongruence may result from inadequate information about the work of the employee. To fit employees' and spouses' perceived employee WFE, supervisors can convey information to spouses by inviting them to the workplace or building information platforms. In addition, supervisors can underline the importance of communication with spouses to employees and serve as role models. Finally, supervisors can offer emotional support to employees in family relationship conflict and buffer the negative effect on emotional exhaustion (Hammer et al., 2009).

Second, from the perspective of employees, our research found that incongruent perceived employees' WFE is associated with higher relationship conflict, especially when spouses' perceived employee WFE is lower than employees' own perception. Employees must learn how to manage spouses' perceived WFE. Managing congruence well in WFE is conducive to both the family and work domains. Communication is an effective method for clarifying the work and family roles of employees (Rizzo et al., 1970). It is particularly important to strengthen communication and foster congruence in perceived WFE. Employees can learn the actual perceived WFE from spouses by communicating with them and adjusting their own perception or that of their spouses accordingly. In addition, employees should be aware that it is detrimental to overestimate their own enrichment to their families.

### Limitations and Future Research

Our research investigated the effect of WFE on the family and work domains from the perspective of the employee–spouse dyad. We contribute to the literature about work–family interfaces by collecting multisource data. In addition, the research was performed in various organizations, ensuring generalizability. Despite these strengths, we acknowledge several limitations that should be considered in future research. First, the data were collected at the same time point. Hence, we cannot assume causality, despite the fact that the findings are consistent with the theoretical rationale. Second, reverse causation may occur between emotional exhaustion and relationship conflict in that emotional exhaustion may spill over from the workplace to the family (e.g., Greenbaum et al., 2014), which can lead spouses to perceive a high level of relationship conflict with employees. Therefore, future studies could utilize a time-lagged design (i.e., family cohesion and emotional exhaustion could be measured 1 month after WFE and relationship conflict are measured) or an experimental design could explore whether spouses' perceived relationship conflict predates employee family cohesion and emotional exhaustion.

Second, our study revealed that the perceptions of spouses regarding the relationship conflict mediated the effects of employee–spouse perceptual congruence in employees' WFE on employee family cohesion and emotional exhaustion on the basis of the conflict theory. Other alternative meditating mechanisms may exist. For example, the fit theory indicates that person–environment/counterpart fit can induce negative sentiment in individuals, which may subsequently impact their attitudes and behaviors (Kristof, 1996; Kristof-Brown et al., 2005). The less aligned an employee's perceived WFE is with that of his/her spouse, the more negative sentiments the supervisor holds toward the subordinate, which, in turn, influences the family cohesion and emotional exhaustion of the employee. Future studies can explore other mediating mechanisms. In addition, we can further extend the scope of our model. We proved that incongruence in WFE can lead to emotional exhaustion of employees in organizations. However, emotional exhaustion may further destroy the positive attitude and behavior of employees. Past research on WFE outcomes has focused largely on job performance, organizational citizenship behavior, organizational commitment, and job satisfaction (e.g., Wayne et al., 2013; Carlson et al., 2014; Qasemi and Behzadi, 2017). Future research can explore the effect of perceived WFE incongruence on the above plausible outcomes.

Third, we paid close attention to the effect of perceived employee WFE (in)congruence while ignoring the variation in the effect under different conditions. Future research can examine boundary conditions. For example, due to the prevalence of gender stereotypes, gender has been regarded as significant in work–family interplay (Ghislieri et al., 2017). Females are typically expected to perform more domestic tasks than males (Heilman, 2012). Compared to the male spouse, the female spouse may be more tolerant of her spouse with low WFE. Hence, for male employees, the impacts of perceived employee WFE (in)congruence on female spouses' perceptions of relationship conflict tend to be weaker. Future research should explore potential boundary conditions, such as gender.

Fourth, our research calculated the “objective” congruence in perceived employee WFE on the basis of employee- and spouse-rated measures. Some research has argued that subjective rather than objective congruence impacts the attitude and performance of employees (Van Vianen et al., 2011). Future research can measure the self-reported perceived congruence of employees or spouses and investigate whether subjective perception has a similar impact on family and work outcomes. In addition, future research can also explore why and when objective and subjective congruence have significantly different impacts.

Fifth, Hypothesis 2 has not been verified in our study. We assumed that the perceptions of the spouse on relationship conflict with the employee are lower when perceived employee WFE is congruent at high levels rather than at low levels. Culture-specific features of work–family interplay may explain why expectation differences are not justified. Work has been considered paramount to family welfare in China. This result indicates that Chinese employees who participate less in family roles will be regarded by spouses as making sacrifices to benefit the family (Redding and Wong, 1986; Redding, 1993). Spouses may be more tolerant and less frustrated when employees have low WFE levels. In Chinese culture, spouses' perceptions of relationship conflict are not necessarily higher when employee WFE is congruent at low levels rather than at high levels. Future studies can replicate our study in different cultural settings.

Finally, although this research has no cultural boundaries, the single Chinese sample may limit the generalizability. Past research has suggested that employees' family members, including spouses, are more likely to be involved in organizations in Chinese culture than in Western culture (Xiao and Cooke, 2012). Employees in Western culture prefer to segment family relationship conflict from work attitude and behavior. It is possible that the effect of WFE (in)congruence on emotional exhaustion is weaker in Western culture. Future studies can examine the research model in different cultural settings.

## Conclusions

Employee WFE has gained much attention from researchers and has been widely believed to positively impact work and family outcomes. In the present research, we take a dyadic approach by incorporating the role of the spouse's perceived employee WFE and examining whether and how employee–spouse perceptual congruence in employee WFE affects employee family and work outcomes. Our findings suggest that it is critical to consider employees' and spouses' perceived employee WFE simultaneously rather than separately, but previous research has usually leaned toward the main impacts of employees' perceived WFE on their own work and family outcomes. It is beneficial to match employees with their spouses in perceived employee WFE. We hope that our investigation will stimulate more research on the potential impacts of employee–spouse perceptual congruence in the work–family interface.

## Data Availability Statement

The raw data supporting the conclusions of this article will be made available by the authors, without undue reservation.

## Ethics Statement

The studies involving human participants were reviewed and approved by Approval was obtained from the ethics committee of Sun Yat-Sen University. The procedures used in this study adhere to the tenets of the Declaration of Helsinki. The patients/participants provided their written informed consent to participate in this study.

## Author Contributions

All authors listed have made a substantial, direct and intellectual contribution to the work, and approved it for publication.

## Conflict of Interest

The authors declare that this study received funding from Shenzhen Shennong Weigu supply chain Co., Ltd. The funder was not involved in the study design, collection, analysis, interpretation of data, the writing of this article or the decision to submit it for publication.
